# Pediatric Borderline Leprosy with Prominent Radial Cutaneous Nerve Enlargement

**DOI:** 10.4269/ajtmh.26-0211

**Published:** 2026-07-09

**Authors:** Harish Kumar Sagar, Harpreet Singh Pawar, Sandeep Sharma

**Affiliations:** ^1^ Clinical Division, Indian Council of Medical Research (ICMR)-National JALMA Institute for Leprosy and Other Mycobacterial Diseases, Agra, India;; ^2^ Indian Council of Medical Research (ICMR)-Head Quarters, New Delhi, India;; ^3^ Field Unit, Indian Council of Medical Research (ICMR)-National JALMA Institute for Leprosy and Other Mycobacterial Diseases Ghatampur, Kanpur, India

*“The first step towards diagnosing leprosy is to think of the possibility of leprosy.”—D. L. Leiker*

The persistence of pediatric leprosy continues to pose a significant challenge to global eradication efforts. Clinical signs are often subtle and require trained health care professionals—particularly in endemic areas—for timely recognition, consideration of diagnosis, and initiation of multidrug therapy.^1^ Conventional invasive diagnostic techniques, such as slit-skin smear (SSS) examination, skin biopsy, and fine-needle aspiration cytology, are usually difficult to perform in children. Therefore, strengthening health care capacity and building clinical expertise in peripheral settings are essential to establish the diagnosis.^2^ Careful clinical evaluation supported by sensitive and specific noninvasive diagnostic tests plays a pivotal role in confirming the disease and ensuring prompt initiation of treatment.

We present the case of a 10-year-old child with wrist pain persisting for 1 month. The pain was severe, radiating from the wrist to the elbow, and it was described as electrifying even on minor touch. Initial evaluation at a nearby primary health center (PHC) suggested a wrist sprain, for which nonsteroidal anti-inflammatory drugs (NSAIDs) were prescribed. Although symptoms temporarily subsided, the pain recurred, and the patient presented to us approximately 3 months after onset.

On clinical examination, an unusual swelling over the right wrist was noted, which was identified as marked thickening of the radial cutaneous nerve. Sensory testing revealed diminished perception, with reduced response to the 2-g monofilament but preserved sensation to the 10-g monofilament, indicating partial loss rather than complete anesthesia along its distribution. The ulnar nerve was also thickened, with sensory deficit but no motor involvement, whereas the median nerve distribution remained intact. These findings were accompanied by a well-defined, dry, hypopigmented patch measuring 5 × 2.5 cm (Figure 1), which was anesthetic to monofilament testing, showing complete loss of sensation to both 2-g and 10-g monofilaments. Other peripheral nerves were unremarkable.

**Figure 1.**
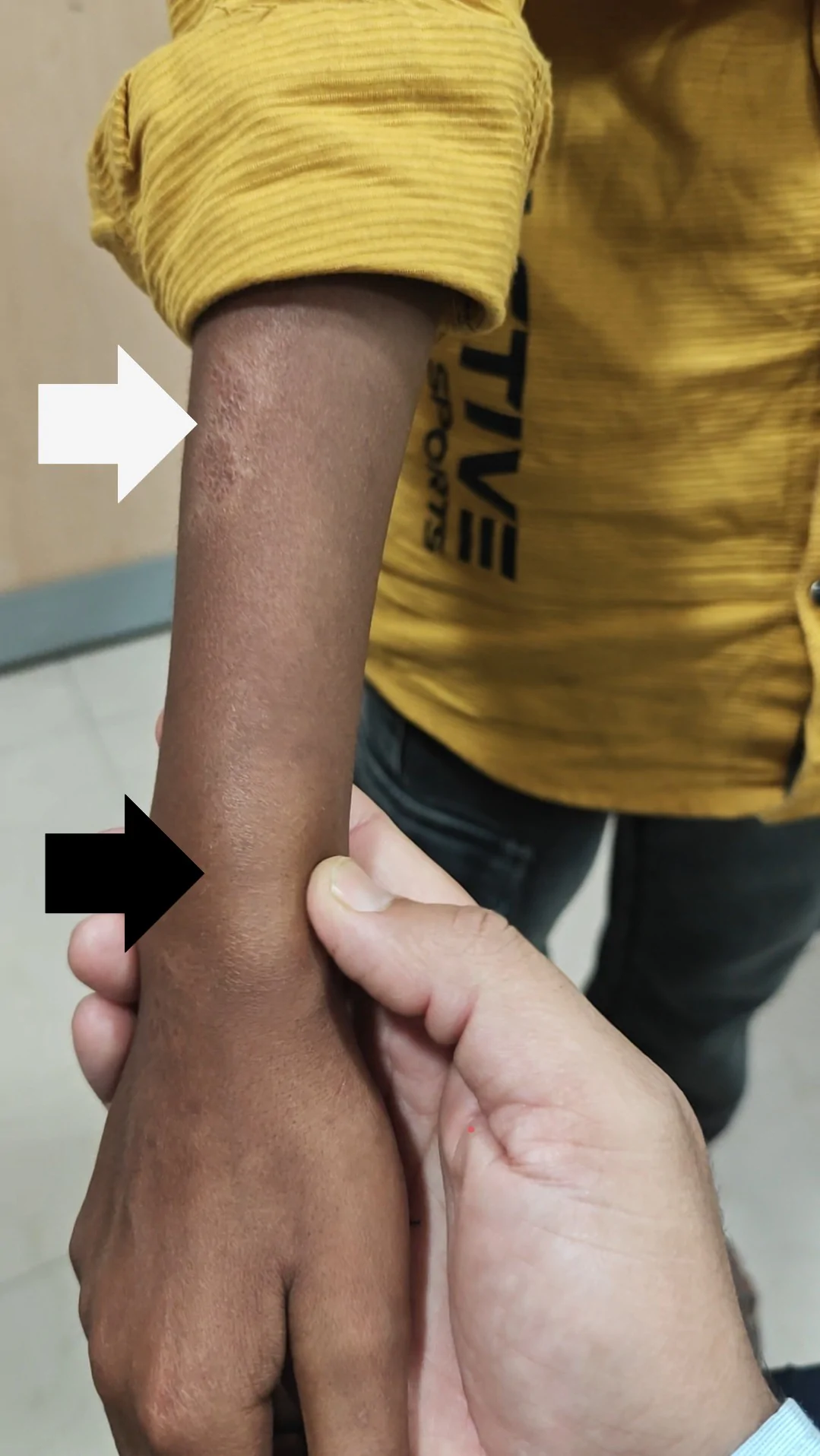
The black arrow points to the enlarged radial cutaneous nerve. The white arrow points to a hypopigmented anesthetic patch.

Nerve conduction studies revealed a sensory-motor axonal damage pattern with reduced sensory conduction velocity and compound motor action potential. Because of heightened sensitivity to pain, the patient declined SSS examination and biopsy. Based on clinical findings and the Ridley–Jopling classification, a diagnosis of borderline leprosy with grade 2 neuritis was established. Multidrug therapy combined with prednisolone (0.6 mg/kg per day) and NSAIDs was initiated, to which the patient responded well. Multiple follow-ups demonstrated progressive improvement.

Pediatric leprosy serves as a critical epidemiological indicator of ongoing transmission and recent disease occurrence in communities, highlighting gaps in existing control programs.^3^ India continues to bear the highest global burden, reporting 100,957 new cases in 2024, including 4,722 among children younger than 15 years old. Compared with other highly endemic countries, India demonstrates a higher prevalence of grade 2 deformities and lower new case detection rates.^4^ Studies have revealed a substantial hidden prevalence of pediatric leprosy in endemic regions, with up to 11% of children presenting with grade 2 disability at diagnosis, which increased to 27.3% during follow-up.^5^^–^^9^ A high rate of misdiagnosis further emphasizes the need to heighten clinical suspicion among health care providers.^10^ Owing to the paucity of noninvasive diagnostic tools, careful clinical evaluation—particularly inspection and peripheral nerve examination—remains indispensable for diagnosis in pediatric and adolescent populations as well as in patients unwilling to undergo invasive procedures. However, advanced noninvasive techniques (nerve conduction velocity/electromyography studies) used for early detection of deformities are not widely available in most PHCs because of high setup costs and limited availability of skilled technicians. Because children are often less cooperative with invasive procedures, health care providers often rely solely on clinical assessment. Inadequate diagnostic skills among existing health care staff members further contribute to delayed diagnosis and progression into deformity. Strengthening health care capacity and emphasizing the development of sensitive, feasible, noninvasive diagnostic approaches are essential for improving case detection. Above all, timely initiation of treatment is paramount as delays can accelerate disease progression, increase deformities, and compromise clinical outcomes.

## Supplemental Materials

10.4269/ajtmh.26-0211 Supplemental Materials
